# Encorafenib plus binimetinib for *BRAF* V600-mutated thyroid cancer in Japan: final analysis

**DOI:** 10.1007/s10147-026-03129-5

**Published:** 2026-07-31

**Authors:** Iwao Sugitani, Naomi Kiyota, Hiroo Imai, Shunji Takahashi, Akihiro Nishiyama, Shingo Tamura, Yasushi Shimizu, Shigenori Kadowaki, Ken-ichi Ito, Yoshinori Hirashima, Shinji Ueno, Teruaki Katayama, Makoto Tahara

**Affiliations:** 1 Department of Endocrine Surgery, Nippon Medical School Hospital, Bunkyo-Ku, Tokyo, 113-8602 Japan; 2 Department of Medical Oncology and Hematology, Cancer Center, Kobe University Hospital, Kobe, Hyogo 650-0017 Japan; 3 Department of Medical Oncology, Tohoku University Hospital, Sendai, Miyagi 980-8575 Japan; 4 Department of Medical Oncology, The Cancer Institute Hospital of JFCR, Koto-Ku, Tokyo, 135-8550 Japan; 5 Department of Medical Oncology, Kanazawa University Hospital, Kanazawa, Ishikawa, 920-0293 Japan; 6 Department of Medical Oncology, NHO Kyushu Medical Center, Fukuoka, Fukuoka, 810-8563 Japan; 7 Department of Medical Oncology, Hokkaido University Hospital, Sapporo, Hokkaido, 060-8648 Japan; 8 Department of Clinical Oncology, Aichi Cancer Center Hospital, Nagoya, Aichi 464-8681 Japan; 9 Division of Breast and Endocrine Surgery, Department of Surgery, Shinshu University School of Medicine, Matsumoto, Nagano, 390-8621 Japan; 10 Ono Pharmaceutical Co., Ltd., Osaka, 541-8564 Japan; 11 Department of Head and Neck Medical Oncology, National Cancer Center Hospital East, Kashiwa, Chiba 277-8577 Japan

**Keywords:** Thyroid cancer, Differentiated thyroid cancer, Anaplastic thyroid cancer, *BRAF* V600E, BRAF inhibitor, MEK inhibitor

## Abstract

**Background:**

The combination of encorafenib plus binimetinib (ENC + BIN) is approved for the treatment of *BRAF* V600-mutated thyroid cancer in Japan, based on a phase 2 trial. We update the efficacy and safety data for ENC + BIN of this phase 2 trial after prolonged follow-up.

**Methods:**

This multicenter, open-label, uncontrolled phase 2 trial evaluated the efficacy and safety of ENC + BIN in patients with unresectable locally advanced or metastatic *BRAF* V600-mutated thyroid cancer that was not amenable to curative treatment, who were refractory/intolerant to ≥ 1 previous vascular endothelial growth factor receptor-targeted regimens, or were considered ineligible for these. The primary endpoint was the objective response rate (ORR). The secondary endpoints included progression-free survival (PFS) and overall survival (OS).

**Results:**

We enrolled 22 patients: 17 with differentiated thyroid carcinoma (DTC) and 5 with anaplastic thyroid carcinoma (ATC). During a prolonged period with a median follow-up of 28.7 months (range: 3.4–40.6), 3 additional patients with DTC achieved partial responses, increasing the ORR to 68.2% (15/22 patients with either DTC or ATC). Medians for PFS and OS were not reached for DTC and 17.7 and 24.3 months, respectively, for ATC. Common adverse events were eye disorder (5/12), infections and infestations (4/12), and skin and subcutaneous tissue disorder (4/12) after > 360 days; no new safety signals were identified during the extended follow-up period.

**Conclusions:**

With a > 24-month median follow-up, this analysis indicates that ENC + BIN provides sustained tumor responses in patients with *BRAF* V600-mutated thyroid cancer. The long-term safety profile is consistent with previous reports.

**Supplementary Information:**

The online version contains supplementary material available at https://doi.org/10.1007/s10147-026-03129-5.

## Introduction

Thyroid cancer mostly originates from thyroid follicular cells and comprises various pathological types of carcinomas, including papillary thyroid carcinoma (PTC), follicular thyroid carcinoma (FTC), oncocytic carcinoma, poorly differentiated thyroid carcinoma, and anaplastic thyroid carcinoma (ATC). PTC and FTC, accounting for about 90% of the thyroid cancer, are well differentiated and are classified as differentiated thyroid cancer (DTC). DTC usually grows slowly and has good treatment outcomes. ATC, on the other hand, is the least common and most aggressive type of thyroid cancer. Mutations and gene fusions drive thyroid cancer development [1–4]. Drugs have been developed targeting key driver mutations and gene fusions, including mutations of *BRAF* and *RET*, and gene fusions of *RET* and *NTRK* [5].

*BRAF* mutation plays an essential role in tumor cell proliferation and survival and is commonly detected in DTC and ATC [2, 6–13]. In Japan, *BRAF* mutations in particular are detected in 74.6% of patients with PTC and in 47.7% of those with ATC [14]. Two combinations of BRAF and MEK inhibitors have been approved for *BRAF*-mutated thyroid cancer in Japan, namely, dabrafenib (DAB) plus trametinib (TRA) [15, 16] and encorafenib (ENC) plus binimetinib (BIN) [17]. In a Japanese phase 2 trial, the ENC + BIN regimen demonstrated clinically meaningful efficacy in terms of the primary endpoint, objective response rate (ORR), in patients with *BRAF* V600-mutated thyroid cancer [17]. ENC is a highly selective BRAF inhibitor with extended pharmacological activity [18, 19], which might lead to better efficacy with prolonged treatment. In the literature, the DAB + TRA regimen has demonstrated a PFS of 15.1 months in patients with DTC [20]; however, the long-term efficacy and safety of the ENC + BIN regimen is still to be determined.

Here, we report the final analysis of the efficacy and safety profile of a phase 2 follow-up study that utilized the ENC + BIN regimen in patients with *BRAF* V600-mutated thyroid cancer.

## Patients and methods

### Patients

Patients with histologically confirmed unresectable locally advanced or distant metastatic thyroid cancer were eligible for the study. Briefly, key inclusion criteria were age ≥ 20 years; thyroid cancer with *BRAF* V600-mutation in tumor tissue or blood samples; refractoriness to or intolerance of 1 or more oral vascular endothelial growth factor receptor (VEGFR)-targeted drugs for thyroid cancer, or those considered to be medically ineligible for those drugs; Eastern Cooperative Oncology Group (ECOG) performance status (PS) of 0 or 1; and the presence of 1 measurable lesion by Response Evaluation Criteria in Solid Tumors (RECIST) v1.1 [21] and a life expectancy of 3 months. Detailed eligibility was reported in the primary paper [17].

The Ethical Review Committee for clinical trials at Ono Pharmaceutical Co., Ltd. conducted the protocol review of this study on October 14, 2020, and approved the trial protocol October 15, 2020. The trial protocol was also approved by the institutional review board at each site. This study was conducted under the Declaration of Helsinki and local regulations. All patients provided written informed consent.

### Study design, treatment and assessments

This multicenter, open-label, uncontrolled phase 2 study (*Japan Registry of Clinical Trials registration No. jRCT2011200018*) aimed to assess the efficacy of the combination of the ENC + BIN regimen in patients with *BRAF* V600-mutated thyroid cancer [17]. Patients orally received ENC (450 mg) once daily and BIN (45 mg) twice daily over a 28-day cycle, until unacceptable toxicity, disease progression, or consent withdrawal occurred. Patients completed the regimen and switched from the trial ENC + BIN regimen to the commercial ENC + BIN regimen after the commercial ENC and BIN became available on May 17, 2024. Drug dose reduction and drug interruption were allowed when the treatment-related adverse events (TRAEs) met prespecified dose adjustment and drug interruption criteria for each drug. The primary endpoint was centrally assessed ORR of overall patients. The secondary endpoints included duration of response (DOR), ratio changes in the size of target tumor during the trial, PFS, and overall survival (OS). All efficacy and safety analyses were conducted by histological classification of thyroid cancer and overall.

Tumor response was assessed according to RECIST v1.1, with assessment for complete response (CR), partial response (PR), stable disease (SD), and progressive disease (PD). Best overall response (BOR) was defined as the best tumor response during the trial. Safety endpoints included AEs and laboratory tests. AE occurrences were assessed until 28 days after the last administration and were graded according to the National Cancer Institute Common Terminology Criteria for Adverse Events v5.0. PFS was defined as the duration from the initiation of treatment to the earliest occurrence of either PD or death from any cause. For PFS analysis, events when that occurred before switching treatment in patients who were assessed as having PD were included. Patients without PD at the time of switching treatment were censored on the last day for which assessable imaging data were available. This rule was applied to patients who switched from the trial ENC + BIN regimen to the same commercial ENC + BIN regimen, as well as to other treatment regimens. DOR was defined as the duration from the earliest day of observation of CR or PR until the earliest occurrence of either PD or death from any cause.

### Statistical analysis

All the data were analyzed for the overall patient group, as well as for the DTC and ATC patient groups. Confidence intervals (CIs) were calculated using the Clopper-Pearson method for ORR and BOR. Medians, CIs, and ratios were calculated using the Kaplan–Meier method for DOR, OS, and PFS. All statistical analysis were performed using Statistical Analysis System software version 9.4 (SAS Institute, Cary, NC, USA).

### Role of funding source

The study sponsor, ONO Pharmaceutical Co., Ltd. (Osaka, Japan), contributed to the study design, as well as operation and supervision of the overall clinical study.

## Results

### Patients

Between March 2021 and May 2022, we enrolled 22 patients (median age, 68) with *BRAF* V600E-mutated thyroid cancer: 17 with DTC and 5 with ATC (Table 1). Of those, 6 (27.3%) and 16 (72.7%) were ECOG PS 0 and 1, respectively. Frequent metastatic sites were lung, lymph nodes, and bone. Twenty patients (90.9%) had a history of VGFR-TKI treatment.

**Table 1:** Patient characteristics

Variable		Overall (n = 22)	DTC (n = 17)	ATC (n = 5)
Sex, n (%)	Male	12 (54.5)	10 (58.8)	2 (40.0)
	Female	10 (45.5)	7 (41.2)	3 (60.0)
Age, years	Median (range)	68.0 (50–77)	67.0 (50–75)	74.0 (60–77)
	< 65	9 (40.9)	8 (47.1)	1 (20.0)
	≥ 65	13 (59.1)	9 (52.9)	4 (80.0)
ECOG PS, n (%)	0	6 (27.3)	6 (35.3)	0
	1	16 (72.7)	11 (64.7)	5 (100)
Initial or recurrent, n (%)	Initial	7 (31.8)	5 (29.4)	2 (40.0)
	Recurrent	15 (68.2)	12 (70.6)	3 (60.0)
Prior therapy, n (%)	Surgery	21 (95.5)	16 (94.1)	5 (100)
	Radiotherapy	8 (36.4)	6 (35.3)	2 (40.0)
	Radioactive therapy (I^131^)	16 (72.7)	15 (88.2)	1 (20.0)
	Thyrotropin suppression therapy	9 (40.9)	7 (41.2)	2 (40.0)
Prior VEGFR-TKI, n (%)	0	2 (9.1)	1 (5.9)	1 (20.0)
	1	16 (72.7)	12 (70.6)	4 (80.0)
	≥2	4 (18.2)	4 (23.5)	0
Duration of prior VEGFR-TKI therapy, months	Median (range)	28.4 (0.3-104.9)	33.4 (1.4–104.9)	10.8 (0.3–52.4)
Metastasis lesion sites, n (%)	Lung	16 (72.7)	12 (70.6)	4 (80.0)
	Lymph nodes	16 (72.7)	13 (76.5)	3 (60.0)
	Bone	6 (27.3)	4 (23.5)	2 (40.0)
	Pleura	5 (22.7)	4 (23.5)	1 (20.0)
	Other	7 (31.8)	5 (29.4)	2 (40.0)
Number of metastases sites, n (%)	< 3	12 (54.5)	9 (52.9)	3 (60.0)
	≥ 3	10 (45.5)	8 (47.1)	2 (40.0)
Sum of diameters of target lesions, mm	Median (range)	40.1 (10.4–132.4)	43.8 (10.4–132.4)	25.6 (13.9–64.0)

Values are shown as n (%) unless otherwise indicated

At the data cutoff (median follow-up, 28.7 months [range: 3.4–40.6]), the median duration of exposure (range) was 17.1 months (2.0–38.8) for ENC and 12.8 months (0.9–38.8) for BIN (Table S1). The median relative dose intensity (range) was 65.1% (18.9–100) for ENC and 64.2% (11.8–100) for BIN. A total of 7 patients (6 with DTC and 1 with ATC) completed the trial ENC + BIN regimen and continued to receive the commercial ENC + BIN regimen (Fig. S1). Treatment discontinuation occurred in 15 patients, due to PD in 12 patients, TRAEs in 2 patients, and consent withdrawal in 1 patient. Twelve patients switched from the trial regimen to other treatments (Table S2).

### Efficacy

During the prolonged follow-up period, 3 additional patients with DTC achieved PR; time to response (TTR) in these 3 patients was 10.1, 10.2, and 13.8 months. The centrally assessed ORR for all patients increased to 68.2% (95% CI 45.1–86.1) (Table 2) from 54.5% in the previous report [17], and the ORR for those with DTC also increased to 64.7% (95% CI 38.3–85.8) from 47.1%. The ORR among patients with ATC remained at 80% (95% CI 28.4–99.5) (See Table S3 for the investigator-assessed ORR). The median TTR (range) was 2.1 months (0.8–13.8) overall, 2.9 months (0.8–13.8) in those with DTC, and 1.4 months (0.9–5.1) in those with ATC (Table 2).

**Table 2:** Objective response rate per central assessment

		Overall (n = 22)	DTC (n = 17)	ATC (n = 5)
ORR	n (%)	15 (68.2)	11 (64.7)	4 (80.0)
	95%CI	45.1-86.1	38.3-85.8	28.4-99.5
BOR	CR	0	0	0
	PR	15 (68.2)	11 (64.7)	4 (80.0)
	SD	7 (31.8)	6 (35.3)	1 (20.0)
	PD	0	0	0
TTR	Median, month	2.1	2.9	1.4
	Range	0.8-13.8	0.8-13.8	0.9-5.1

BOR, Best overall response; ORR, Objective response rate; TTR, Time to response

**Fig. 1:**
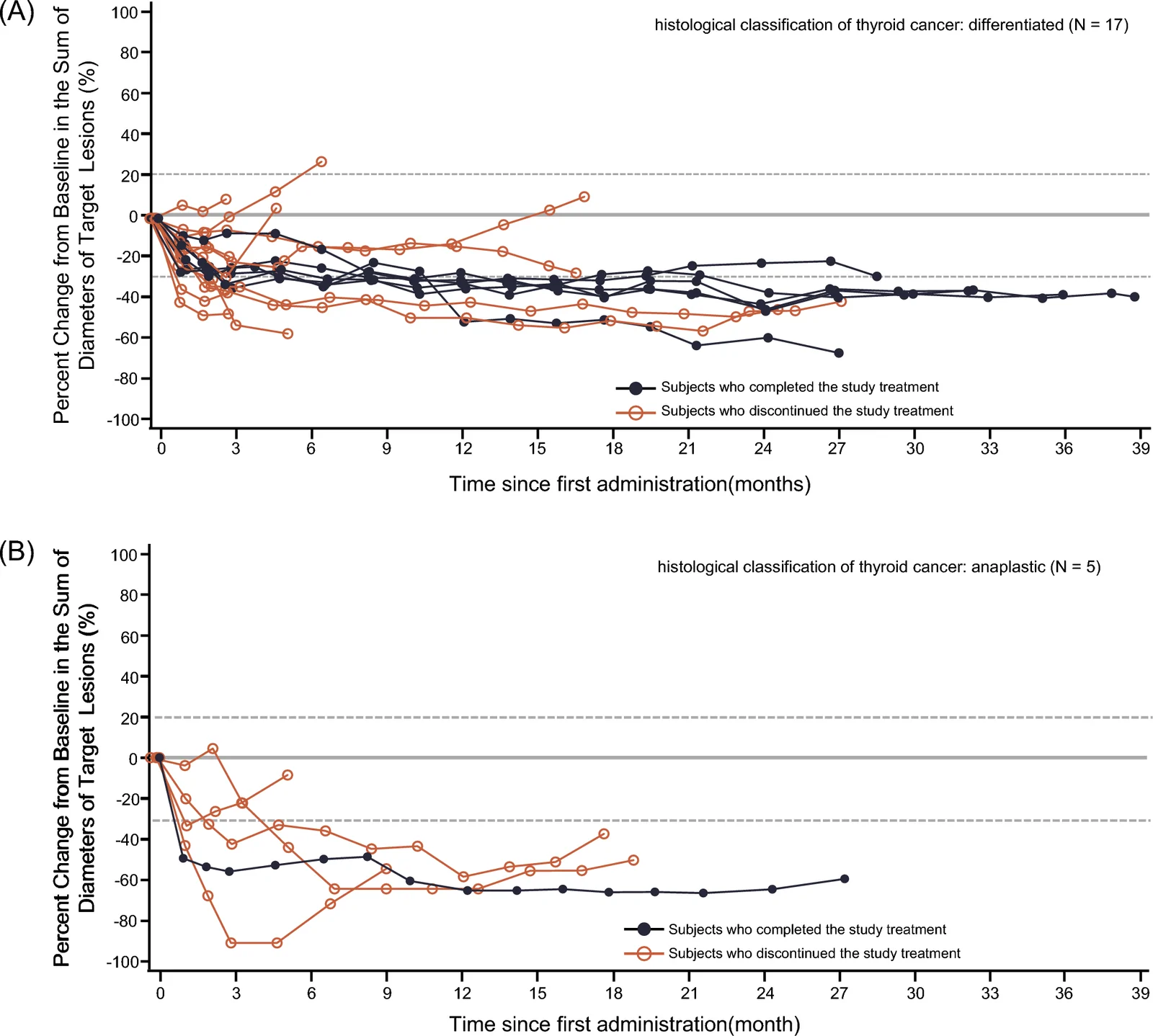
Spider plot. Spider plot showing percent change from baseline in the sum of diameters of target lesions of patient with DTC (**A**) and those with ATC (**B**). The plots include patients with target lesions at baseline and post-baseline tumor assessment (DTC: 17, ATC: 5). Blue and orange lines indicate subjects who completed the study treatment and discontinued the study treatment, respectively

**Fig. 2:**
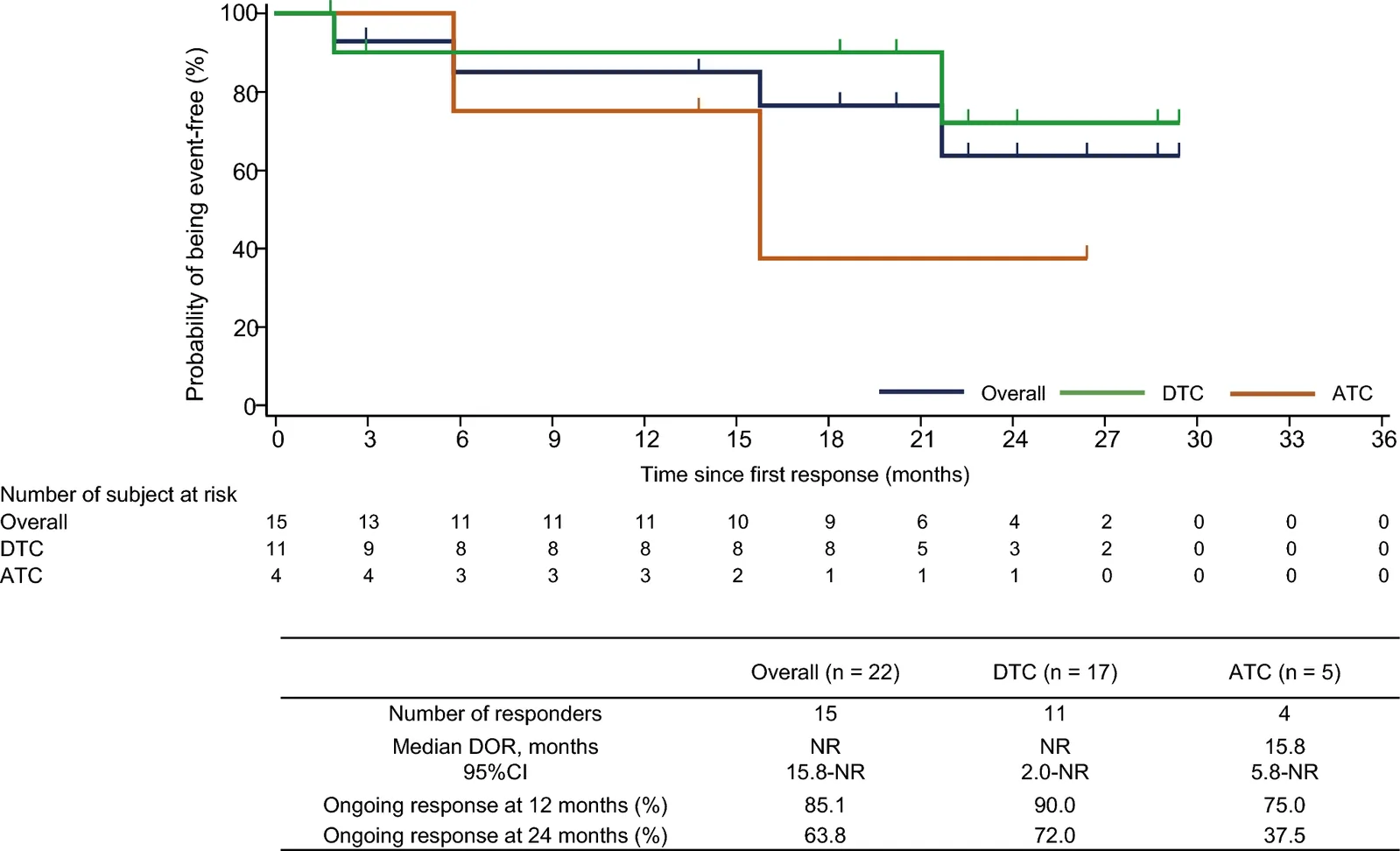
Duration of response (DOR). Kaplan–Meier curves showing DOR based on central assessment for all patients (blue), those with DTC (green), and those with ATC (orange). The table shows statistics for DOR. NR, not reached

**Fig. 3:**
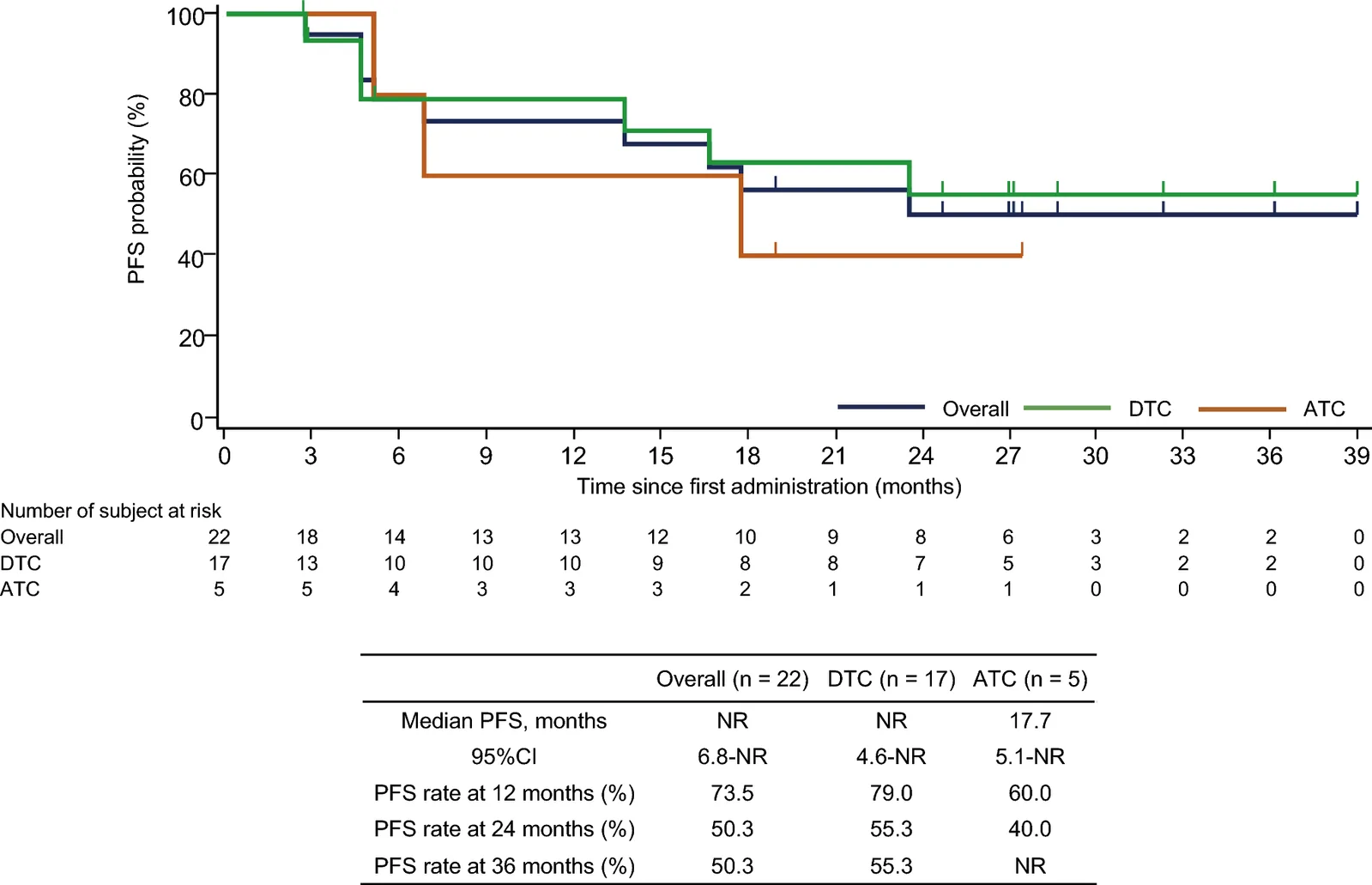
Progression free survival (PFS). Kaplan–Meier curves showing PFS based on central assessment for all patients (blue), those with DTC (green), and those with ATC (orange). The table shows statistics for PFS. NR, not reached

**Fig. 4:**
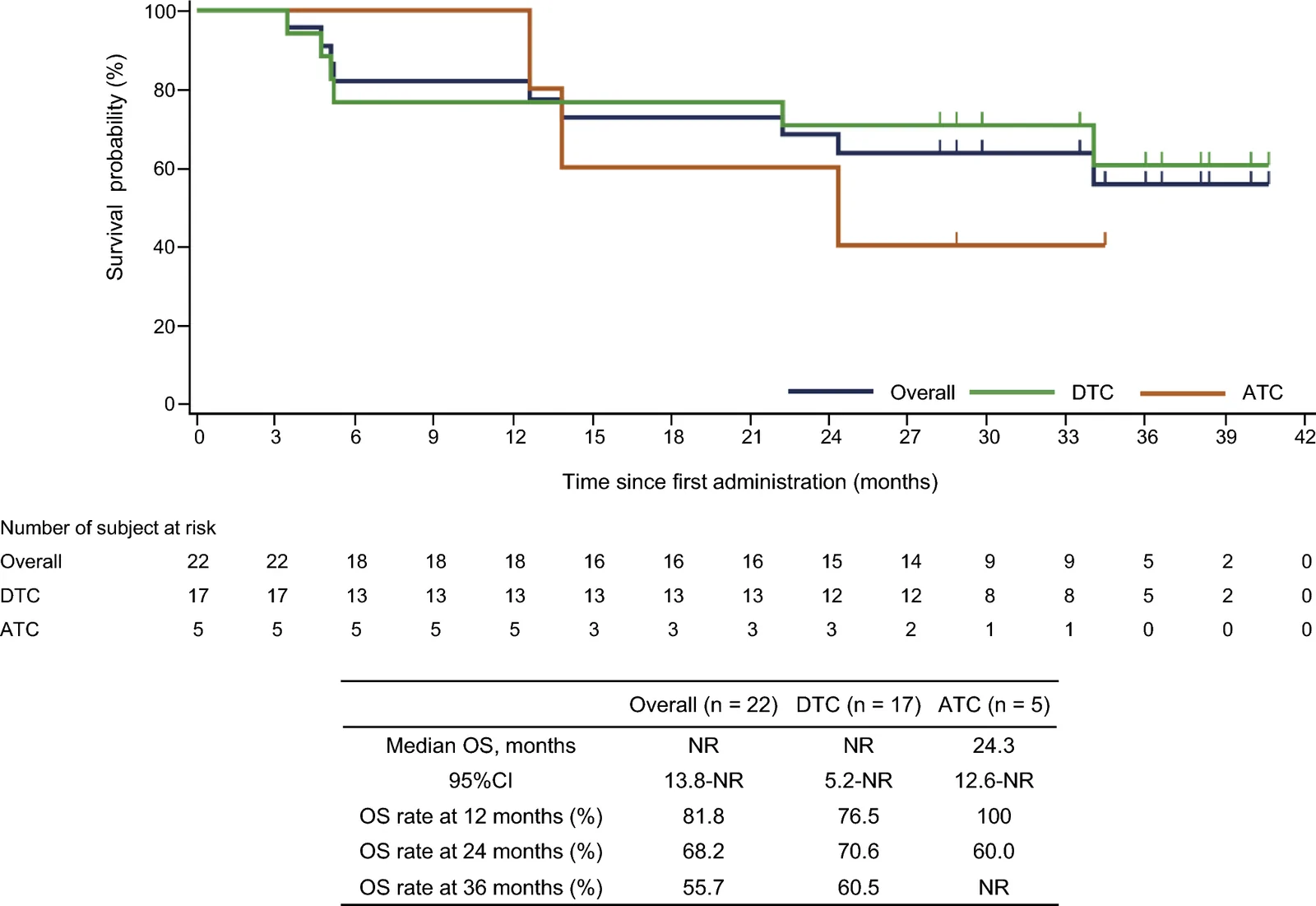
Overall survival (OS). Kaplan–Meier curves showing OS for all patients (blue), those with DTC (green), and those with ATC (orange). The table shows statistics for OS. NR, not reached

Most patients showed size reduction with respect to the best change from baseline in the target tumor (21/22 in overall patients) (Fig. S2). During the prolonged follow-up period, tumor size reduction was continuously observed (Fig. 1), resulting in further decreases in target tumor of 8 patients with DTC and 1 with ATC (Fig. S2). The median DOR was not reached in patients overall, nor in those with DTC; the value was 15.8 months in patients with ATC (Fig. 2). The median PFS was not reached in patients with DTC, but was 17.7 months with ATC (Fig. 3). PFS rates at 24 months were 55.3% in patients with DTC and 40.0% in those with ATC. The OS rate was still beyond 50% in patients with DTC, and the median OS was 24.3 months with ATC (Fig. 4).

#### Safety

AEs of any grade and grade 3–4 occurred in 22 (100%) and 8 (36.4%), respectively; no grade 5 AEs were reported (Table 3). Common grade 3–4 AEs were lipase increased (4 patients, 18.2%). Two grade 4 AEs, glaucoma and acute kidney injury, were reported; however, a relationship between these AEs and the trial regimen was ruled out. Frequent AEs were nausea (11 patients, 50.0%), palmar-plantar erythrodysaesthesia syndrome (7 patients, 31.8%), and serous retinal detachment (7 patients, 31.8%) (Table 4). While a significant fraction of the AEs (71.1%) occurred within the 90 days of treatment initiation, several AEs were observed more than 360 days post treatment initiation, such as eye disorders (5 patients, 41.7%), infections and infestations (4 patients, 33.3%), and skin and subcutaneous tissue disorder (4 patients, 33.3%); however, no new safety signals were identified during the prolonged follow-up period (Fig. S3 and Table S4).

**Table 3:** Summary of safety

(A)						
	Overall (n = 22)		DTC (n = 17)		ATC (n = 5)	
	Any grade	Grade 3/4	Any grade	Grade 3/4	Any grade	Grade 3/4
*Adverse events (AEs), n (%)*	**22 (100)**	**8 (36.4)**	**17 (100)**	**6 (35.3)**	**5 (100)**	**2 (40.0)**
Serious AEs	4 (18.2)	3 (13.6)	2 (11.8)	2 (11.8)	2 (40.0)	1 (20.0)
*Treatment related AEs (TRAEs), n (%)*	**21 (95.5)**	**7 (31.8)**	**16 (94.1)**	**5 (29.4)**	**5 (100)**	**2 (40.0)**
Serious TRAEs	1 (4.5)	1 (4.5)	0	0	1 (20.0)	1 (20.0)
TRAEs dose interruption of any study treatment	19 (86.4)	4 (18.2)	14 (82.4)	3 (17.6)	5 (100)	1 (20.0)
TRAEs dose reduction of any study treatment	3 (13.6)	0	2 (11.8)	0	1 (20.0)	0
TRAEs discontinuation of any study treatment*	4 (18.2)	2 (9.1)	4 (23.5)	2 (11.8)	0	0
TRAEs leading death	0	0	0	0	0	0

(B)		
Patients	TRAE (Grade)	Outcome
No. 1	Ejection fraction decreased (Grade 2)	Resolving
No. 2	Ejection fraction decreased (Grade 2)	Resolving
No. 3	Allergic reaction to investigational drug (Grade 3)	Resolved
No. 4	Ejection fraction decreased (Grade 2)	Resolved
	Palmar-plantar erythrodysaesthesia syndrome (Grade 3)	Resolving

^*^ indicates that all discontinuation TRAEs were resolved or resolving

AE occurrences were assessed until 28 days after the last administration

**Table 4:** Common adverse events (incidence rate ≥ 10%)

System organ class	Overall (n = 22)	
Preferred term	Any grade	Grade 3/4
Any adverse event	**22 (100)**	**8 (36.4)**
Skin and subcutaneous tissue disorders	**18 (81.8)**	**3 (13.6)**
Palmar-plantar erythrodysaesthesia syndrome	7 (31.8)	1 (4.5)
Pruritus	5 (22.7)	1 (4.5)
Rash maculo-papular	5 (22.7)	0
Rash	4 (18.2)	0
Gastrointestinal disorders	**17 (77.3)**	**2 (9.1)**
Nausea	11 (50.0)	0
Diarrhoea	5 (22.7)	1 (4.5)
Vomiting	5 (22.7)	0
Constipation	3 (13.6)	0
Stomatitis	3 (13.6)	0
Eye disorders	**17 (77.3)**	**1 (4.5)**
Serous retinal detachment	7 (31.8)	0
Macular oedema	4 (18.2)	0
Visual field defect	3 (13.6)	0
Infections and infestations	**13 (59.1)**	**0**
COVID-19	5 (22.7)	0
Nasopharyngitis	4 (18.2)	0
General disorders and administration site conditions	**11 (50.0)**	**0**
Pyrexia	6 (27.3)	0
Fatigue	5 (22.7)	0
Musculoskeletal and connective tissue disorders	**10 (45.5)**	**0**
Arthralgia	6 (27.3)	0
Myalgia	4 (18.2)	0
Metabolism and nutrition disorders	**9 (40.9)**	**0**
Decreased appetite	6 (27.3)	0
Hyperuricaemia	3 (13.6)	0
Endocrine disorders	**3 (13.6)**	**0**
Hypothyroidism	3 (13.6)	0
Investigations	**16 (72.7)**	**6 (27.3)**
Blood creatine phosphokinase increased	5 (22.7)	0
Blood creatinine increased	5 (22.7)	0
Ejection fraction decreased	4 (18.2)	0
Lipase increased	4 (18.2)	4 (18.2)
Gamma-glutamyl transferase increased	3 (13.6)	1 (4.5)
Neutrophil count decreased	3 (13.6)	2 (9.1)

Summary of system organ class with at least 1 preferred term having incidence of 10% or more. Values are shown as n (%) unless otherwise indicated. AE occurrences were assessed until 28 days after the last administration

TRAEs of either ENC or BIN occurred in 21 patients (95.5%) for any grade, 7 (31.8%) for grade 3–4, and 0 for grade 5 (Table 3). A serious TRAE, duodenal ulcer, was observed in 1 patient with ATC and recovered with treatment discontinuation and treatment with a proton pump inhibitor. Four patients discontinued the trial treatment due to the following TRAEs: grade 2 ejection fraction decreased (3 cases), although none experienced symptomatic heart failure; grade 3 palmar-plantar erythrodysaesthesia syndrome (1 case); and grade 3 allergic reaction to the investigational drug (1 case) (There was a redundancy in the TRAEs for these dose reductions. See Table 3 for detail). Table S5 summarizes frequent TRAEs during the trial period. Most of these eye disorders (29/32 events) recovered or were recovering; 3 unresolved events were grade 1 serous retinal detachment in 2 cases and grade 1 eye disorder in 1, and all patients continued trial treatment (Table S6). Overall, most of the patients with TRAEs, including those with serous retinal detachment and lipase increased, had recovered or were recovering with appropriate intervention, such as drug interruption (Table S7).

## Discussion

This final analysis of the phase 2 study evaluated the long-term efficacy and safety of the ENC + BIN regimen in patients with *BRAF*-mutated thyroid cancer that was not amenable to curative treatment. The centrally assessed ORRs were 68.2% in the overall population, 64.7% in those with DTC, and 80% in those with ATC; during the extended follow-up period, 3 additional patients with DTC achieved PR. During the prolonged follow-up period, no new safety signals were identified.

Median TTR with ENC + BIN was 2.1 months for patients with thyroid cancer in this study and 1.9 months for patients with melanoma in the COLUMBUS study [22]. These short TTRs are consistent with the general observation that molecular targeted agents tend to exert their efficacy within a short period. Notably, prolonged treatment with ENC + BIN induced tumor responses at later time points: 3 of 17 patients with DTC achieved PR after more than 10 months of treatment in this study. Similarly, DAB + TRA regimen has been reported to induce delayed responses, with 1 of 27 patient with DTC achieving a minor response (defined as a 20–30% reduction in tumor size) after 6–12 months of treatment with different combination of DAB + TRA [20]. In contrast, patients with melanoma treated with the same combination of ENC + BIN have not shown a tendency toward delayed tumor responses [23, 24], suggesting that such late responses in thyroid cancer may result from a unique tumor biology, possibly involving the stepwise acquisition of additional mutations driving malignancy.

PTC enhances its malignant tendencies by acquiring mutations in the *TERT* promoter in addition to *BRAF* mutation; sequential acquisition drives anaplastic transformation of PTC through stepwise accumulation of additional mutations, such as those in *TP53* [1, 10]. Consistently, the Cancer Genome Atlas (TCGA) project has revealed the following features of *BRAF*-mutated PTC: proliferation activity varies among tumor cells, and these differences are largely dependent on activation of the BRAF-MEK-ERK signaling axis. These findings suggest that tumor cells progressively enhance their malignancy through stepwise mutation acquisition associated with increasing activation of the BRAF-MEK-ERK pathway over time [1]. Interestingly, combinations of BRAF and MEK inhibitors provides better ORRs in ATC than in DTC [15, 17, 20]. Taken together, our hypothesis is that DTC gradually acquires higher malignant potential through progressive activation of the BRAF-MEK-ERK axis driven by accumulating mutations. Paradoxically, the ENC + BIN regimen effectively suppresses these more aggressive DTC by directly inhibiting the same pathway, which may account for the delayed yet promising tumor responses observed in this study. These hypothetical interpretations remain speculative and warrant further pathological and genetic validation in future investigations.

With a median follow-up of 28.7 months, this study provided promising PFS and DOR: the median PFS and DOR of the ENC + BIN regimen for DTC were not reached (PFS rate was 55.3% and DOR rate was 72.0% at 24 months), whereas for ATC they were 17.7 months and 15.8 months, respectively. These promising tumor responses of the ENC + BIN regimen, including PFS, DOR, and ORR, during prolonged follow-up period, can partly result from an exceptionally long dissociation half-life of ENC (> 30 h) [18], which may lead to sustained BRAF inhibition even after plasma clearance. Meanwhile, 7 patients completed the trial treatment and transitioned to commercial ENC + BIN treatment, which may have led to underestimation of PFS and DOR and may also have introduced the possibility of informative censoring. While informative censoring cannot be completely ruled out, cases with confirmed PD before the transition were included as events in the PFS and DOR analyses, reducing the impact of censoring due to switching therapy. This promising efficacy of ENC + BIN should be further confirmed in future studies with a larger sample size and longer follow-up, given the uncontrolled design of this trial, limited sample size (particularly for patients with ATC), and immaturity of the PFS and DOR data.

This final follow-up evaluated long-term safety of the ENC + BIN regimen. Although patients experienced some AEs more than 360 days after treatment initiation, no new safety signals were identified during the prolonged study period compared with previous studies involving ENC + BIN [17, 22–24]. Eye disorders are class effect of MEK inhibitors and are typically reported at the early stage of treatment [17, 25, 26]. In this study, two cases of eye disorders were also reported during longer follow-up (more than 90 days from treatment initiation); however, most events during the entire observation period had resolved or were resolving, consistent with previous clinical trials [17, 25]. These results suggest that eye disorders can be effectively managed with appropriate intervention, including dose control. Ejection fraction decreased led to treatment discontinuation in one patient in this study. This event is also a class effect of MEK inhibitors and is thought to result from transient cardiomyocyte dysfunction. Ejection fraction decreased occurs infrequently (5–11%) and is generally reversible with a favorable prognosis in patients with melanoma [22, 27, 28]. Therefore, cardiac function was regularly monitored by ultrasound cardiography (during the screening period and on start day of each cycle after 2nd cycle) during the trial period. All events related to ejection fraction decreased in this study had resolved or were resolving, consistent with previous reports [22, 27, 28], suggesting the importance of regular monitoring for ejection fraction decreased. Taken together, the regimen’s safety concerns could be managed with appropriate management, such as dose reduction for ocular toxicities and regular monitoring of ejection fraction. These appropriate safety management may support the benefit of long-term treatment with ENC + BIN in patients with *BRAF*-mutated thyroid cancer. However, further analyses on safety profile with a larger sample size and longer follow-up are warranted to characterize safety profile precisely, given the limited sample size of this study.

This study has several limitations. One limitation is the absence of a control group, which restricts the interpretation of the regimen’s efficacy. Another limitation is potential selection bias and small sample size due to the rarity of the target disease, although the sample size met the predefined requirements. A future trial with an appropriate control group and a larger sample size will help address these limitations. Although PD events were appropriately counted prior to censoring, the potential impact of informative censoring related to treatment switching should be considered, when interpreting the Kaplan–Meier analysis. Lastly, *BRAF* V600 mutation testing was performed only during the screening period; therefore, the relationship between stepwise mutation acquisition and the efficacy of the trial regimen remains unclear. This also represents an important direction for future research.

With a median follow-up of > 24 months, this analysis suggests that the ENC + BIN regimen may provide durable tumor responses in patients with *BRAF* V600-mutated thyroid cancer. The long-term safety profile of the regimen is consistent with that observed in previous studies.

## Supplementary Information

Below is the link to the electronic supplementary material.

Supplementary Material 1

## Data Availability

Any researcher may request Ono Pharmaceutical Co., Ltd. to disclose individual patient‐level data from clinical studies through Vivli (https://vivli.org/). Interested researchers should consult https://www.ono-pharma.com/en/company/policies/clinical_trial_data_transparency_policy.html for more information on Ono Pharmaceutical Co., Ltd.’s Global Policy about the Disclosure of Clinical Study Data.
