# Supplementary material for: Encorafenib plus binimetinib for BRAF V600-mutated thyroid cancer in Japan: final analysis
Source: Int J Clin Oncol. 2026 Jul 31;31(10):2223–33. doi: 10.1007/s10147-026-03129-5 (PMC13615043; doi:10.1007/s10147-026-03129-5)
Supplement: Supplementary file 1 [file 10147_2026_3129_MOESM1_ESM.docx]

Supplementary Figure Legends

Figure S1. Subject disposition for patients with DTC and ATC in the trial

* indicates that 7 patients (DTC: 6, ATC: 1) continued to receive commercial encorafenib plus binimetinib regimen.

Figure S2. Waterfall plot

Waterfall plot showing the maximum percent reduction from baseline in patients with either DTC (green) or ATC (orange). The plot includes patients with target lesion at baseline and post-baseline tumor assessment (DTC:17, ATC: 5). † indicates further decrease in target tumor during the prolonged follow-up period compared to the previous report [17].

Figure S3. Time to onset for adverse events (AEs)

Bar graph showing the number of patients who developed the specific AEs (system organ class with at least 1 preferred term having an incidence of 10% or more) during the indicated period.

Supplementary Tables

Table S1: Exposure of study treatment

|  | **Overall (n = 22)** | | **DTC (n = 17)** | | **ATC (n = 5)** | |
| --- | --- | --- | --- | --- | --- | --- |
|  | **Encorafenib** | **Binimetinib** | **Encorafenib** | **Binimetinib** | **Encorafenib** | **Binimetinib** |
| **Duration of exposure, months** |  |  |  |  |  |  |
| Median | 17.1 | 12.8 | 16.6 | 7.3 | 17.7 | 17.7 |
| Range | 2.0-38.8 | 0.9-38.8 | 2.0-38.8 | 0.9-38.8 | 5.6-27.3 | 5.6-27.3 |
| **Relative dose intensity** |  |  |  |  |  |  |
| Median | 65.1 | 64.2 | 71.8 | 66.5 | 55.7 | 55.5 |
| Range | 18.9-100 | 11.8-100 | 18.9-100 | 11.8-100 | 47.1-68.3 | 31.5-65.2 |

Table S2: Subsequent therapy

|  | **Overall (n = 22)** | **DTC (n = 17)** | **ATC (n = 5)** |
| --- | --- | --- | --- |
| **Any subsequent therapy, n (%)** | **12* (54.5)** | **8 (47.1)** | **4 (80.0)** |
| Lenvatinib | 7 (31.8) | 4 (23.5) | 3 (60.0) |
| Sorafenib | 3 (13.6) | 3 (17.6) | 0 |
| Radiotherapy | 2 (9.1) | 1 (5.9) | 1 (20.0) |
| * indicates that 12 patients switched to the next treatment due to disease progression (9 patients), adverse events (2), or consent withdrawal (1). | | | |

Table S3: Objective response rate per investigator assessment

|  | | **Overall (n = 22)** | **DTC (n = 17)** | **ATC (n = 5)** |
| --- | --- | --- | --- | --- |
| **ORR** | **n (%)** | 14 (63.6) | 10 (58.8) | 4 (80.0) |
|  | **95%CI** | 40.7-82.8 | 32.9-81.6 | 28.4-99.5 |
| **BOR** | **CR** | 1 (4.5) | 0 | 1 (20.0) |
|  | **PR** | 13 (59.1) | 10 (58.8) | 3 (60.0) |
|  | **SD** | 8 (36.4) | 7 (41.2) | 1 (20.0) |
|  | **PD** | 0 | 0 | 0 |
| Abbreviations: BOR, best overall response; ORR, objective response rate | | | | |

Table S4: Common adverse events more than 360 days after treatment initiation (incidence rate ≥10%)

| **System Organ Class** | **Overall (n = 12)** | |
| --- | --- | --- |
| Preferred Term | **Any grade** | **Grade 3/4** |
|  | **n (%)** | **n (%)** |
| **Eye disorders** | **5 (41.7)** | **1 (8.3)** |
| Serous retinal detachment | 2 (16.7) | 0 |
| **Infections and infestations** | **4 (33.3)** | **0** |
| Nasopharyngitis | 2 (16.7) | 0 |
| **Skin and subcutaneous tissue disorders** | **4 (33.3)** | **0** |
| **Gastrointestinal disorders** | **3 (25.0)** | **0** |
| Nausea | 2 (16.7) | 0 |
| **General disorders and administration site conditions** | **2 (16.7)** | **0** |
| **Immune system disorders** | **2 (16.7)** | **0** |
| **Metabolism and nutrition disorders** | **2 (16.7)** | **0** |
| Hyperuricaemia | 2 (16.7) | 0 |
| **Respiratory, thoracic and mediastinal disorders** | **2 (16.7)** | **0** |
| **Investigations** | **5 (41.7)** | **0** |
| Summary of system organ class having an incidence of 10% or more. Adverse events were assessed in 12 patients who continued the trial treatment for more than 360 days. Adverse events occurrences were assessed until 28 days after the last administration. | | |

Table S5: Common treatment-related adverse events (incidence rate ≥10%)

| **System Organ Class** | **Overall (n = 22)** | |
| --- | --- | --- |
| Preferred Term | **Any grade** | **Grade 3** |
| **Any adverse event, n (%)** | **21 (95.5)** | **7 (31.8)** |
|  |  |  |
| **Eye disorders** | **17 (77.3)** | **0** |
| Serous retinal detachment | 7 (31.8) | 0 |
| Macular oedema | 3 (13.6) | 0 |
| Visual field defect | 3 (13.6) | 0 |
| **Skin and subcutaneous tissue disorders** | **14 (63.6)** | **2 (9.1)** |
| Palmar-plantar erythrodysaesthesia syndrome | 7 (31.8) | 1 (4.5) |
| Rash maculo-papular | 4 (18.2) | 0 |
| Pruritus | 3 (13.6) | 1 (4.5) |
| Rash | 3 (13.6) | 0 |
| **Gastrointestinal disorders** | **13 (59.1)** | **2 (9.1)** |
| Nausea | 10 (45.5) | 0 |
| Diarrhoea | 4 (18.2) | 1 (4.5) |
| Vomiting | 4 (18.2) | 0 |
| Stomatitis | 3 (13.6) | 0 |
| **General disorders and administration site conditions** | **8 (36.4)** | **0** |
| Fatigue | 5 (22.7) | 0 |
| **Musculoskeletal and connective tissue disorders** | **7 (31.8)** | **0** |
| Arthralgia | 4 (18.2) | 0 |
| Myalgia | 3 (13.6) | 0 |
| **Metabolism and nutrition disorders** | **7 (31.8)** | **0** |
| Decreased appetite | 6 (27.3) | 0 |
| **Endocrine disorders** | **3 (13.6)** | **0** |
| Hypothyroidism | 3 (13.6) | 0 |
| **Investigations** | **14 (63.6)** | **6 (27.3)** |
| Blood creatine phosphokinase increased | 4 (18.2) | 0 |
| Blood creatinine increased | 4 (18.2) | 0 |
| Ejection fraction decreased | 4 (18.2) | 0 |
| Lipase increased | 4 (18.2) | 4 (18.2) |
| Gamma-glutamyl transferase increased | 3 (13.6) | 1 (4.5) |
| Summary of system organ class with at least 1 preferred term having an incidence of 10% or more. Adverse events occurrences were assessed until 28 days after the last administration. | | |

Table S6: Outcomes of TRAEs related to eye disorders

| **System Organ Class** |  | **Outcome, n (%)** | | |
| --- | --- | --- | --- | --- |
| Preferred Term | **Events, n** | **Recovered** | **Recovering** | **Unrecovered** |
| **Eye disorders** | **32** | **28 (87.5)** | **1 (3.1)** | **3* (9.4)** |
| Serous retinal detachment | 14 | 12 (85.7) | 0 | 2 (14.3) |
| Macular oedema | 3 | 2 (66.7) | 1 (33.3) | 0 |
| Visual field defect | 3 | 3 (100) | 0 | 0 |
| Visual impairment | 3 | 3 (100) | 0 | 0 |
| Retinopathy | 2 | 2 (100) | 0 | 0 |
| Cataract | 1 | 1 (100) | 0 | 0 |
| Detachment of retinal pigment epithelium | 1 | 1 (100) | 0 | 0 |
| Eye disorder | 1 | 0 | 0 | 1 (100) |
| Photopsia | 1 | 1 (100) | 0 | 0 |
| Retinal exudates | 1 | 1 (100) | 0 | 0 |
| Subretinal fluid | 1 | 1 (100) | 0 | 0 |
| Vitreous opacities | 1 | 1 (100) | 0 | 0 |
| * indicates that all three unrecovered events were categorized as Grade 1.  AE occurrences were assessed until 28 days after the last administration. There were no patients who experienced sequelae following recovery and no cases of death or unknown outcomes. | | | | |

Table S7: Outcomes of major TRAEs (incidence rate ≥10%)

| **System Organ Class** |  | **Outcome, n (%)** | | |
| --- | --- | --- | --- | --- |
| Preferred Term | **Patients, n** | **Recovered** | **Recovering** | **Unrecovered** |
| **Eye disorders** | **17** | **13 (76.5)** | **1 (5.9)** | **3 (17.6)** |
| Serous retinal detachment | 7 | 5 (71.4) | 0 | 2 (28.6) |
| Macular oedema | 3 | 2 (66.7) | 1 (33.3) | 0 |
| Visual field defect | 3 | 3 (100) | 0 | 0 |
| **Skin and subcutaneous tissue disorders** | **14** | **4 (28.6)** | **3 (21.4)** | **7 (50.0)** |
| Palmar-plantar erythrodysaesthesia syndrome | 7 | 3 (42.9) | 2 (28.6) | 2 (28.6) |
| Rash maculo-papular | 4 | 2 (50.0) | 0 | 2 (50.0) |
| Pruritus | 3 | 2 (66.7) | 1 (33.3) | 0 |
| Rash | 3 | 2 (66.7) | 0 | 1 (33.3) |
| **Gastrointestinal disorders** | **13** | **11 (84.6)** | **0** | **2 (15.4)** |
| Nausea | 10 | 9 (90.0) | 0 | 1 (10.0) |
| Diarrhoea | 4 | 3 (75.0) | 0 | 1 (25.0) |
| Vomiting | 4 | 4 (100) | 0 | 0 |
| Stomatitis | 3 | 3 (100) | 0 | 0 |
| **General disorders and administration site conditions** | **8** | **5 (62.5)** | **1 (12.5)** | **2 (25.0)** |
| Fatigue | 5 | 4 (80.0) | 0 | 1 (20.0) |
| **Metabolism and nutrition disorders** | **7** | **7 (100)** | **0** | **0** |
| Decreased appetite | 6 | 6 (100) | 0 | 0 |
| **Musculoskeletal and connective tissue disorders** | **7** | **4 (57.1)** | **0** | **3 (42.9)** |
| Arthralgia | 4 | 2 (50.0) | 0 | 2 (50.0) |
| Myalgia | 3 | 3 (100) | 0 | 0 |
| **Endocrine disorders** | **3** | **0** | **2 (66.7)** | **1 (33.3)** |
| Hypothyroidism | 3 | 0 | 2 (66.7) | 1 (33.3) |
| **Investigations** | **14** | **10 (71.4)** | **2 (14.3)** | **2 (14.3)** |
| Blood creatine phosphokinase increased | 4 | 4 (100) | 0 | 0 |
| Blood creatinine increased | 4 | 3 (75.0) | 0 | 1 (25.0) |
| Ejection fraction decreased | 4 | 2 (50.0) | 2 (50.0) | 0 |
| Lipase increased | 4 | 3 (75.0) | 1 (25.0) | 0 |
| Gamma-glutamyl transferase increased | 3 | 2 (66.7) | 0 | 1 (33.3) |
| Summary of system organ class with at least 1 preferred term having an incidence of 10% or more. AE occurrences were assessed until 28 days after the last administration. There were no patients who experienced sequelae following recovery and no cases of death or unknown outcomes. | | | | |
